# Effect of different dietary patterns on patients with type 2 diabetes: a systematic review and meta-analysis of randomized controlled trials

**DOI:** 10.3389/fnut.2026.1929818

**Published:** 2026-10-12

**Authors:** Haojie Xu, Haotian Ma, Siyao Jin, Yanming Li, Shaolong Zhang, Libo Zhao

**Affiliations:** 1 Department of Pharmacy, Peking University Third Hospital, Beijing, China; 2 Department of Pharmacy Administration and Clinical Pharmacy, Peking University, Beijing, China; 3 Department of Pharmacy, Beijing Children's Hospital, Capital Medical University, Beijing, China; 4 Therapeutic Drug Monitoring and Clinical Toxicology Center, Peking University, Beijing, China

**Keywords:** diet, glycemic control, ketogenic diet, Mediterranean diet, systematic review, type 2 diabetes

## Abstract

**Background:**

Type 2 diabetes mellitus (T2DM) affects over 500 million people worldwide, and dietary management is a cornerstone of treatment. This meta-analysis descriptively evaluated the comparative efficacy and safety of four common dietary patterns—Mediterranean diet (MD), intermittent fasting (IF), energy-restricted diet (ERD), and ketogenic diet (KD)—in adults with T2DM.

**Methods:**

**\**We searched the Cochrane Library, Embase, PubMed, and Web of Science from inception to March 13, 2025, for randomized controlled trials (RCTs). Outcomes included glycemic control (FBG, HbA1c), anthropometric parameters (weight, BMI, waist circumference), lipid profiles (TC, LDL-C, TG), and safety data. Random-effects models were used, with heterogeneity assessed by the I^2^ statistic, and sensitivity analyses via leave-one-out. Risk of bias was evaluated using the Cochrane RoB 2.0 tool.

**Results:**

A total of 25 RCTs were included. Reductions in FBG (MD = −0.63; I^2^ = 62.1%) and HbA1c (MD = −0.50; I^2^ = 83.8%) were observed, but the pooled estimates were limited by substantial heterogeneity and prediction intervals crossing zero. The MD showed excellent cross-study consistency for FBG (I^2^ = 0%) with its prediction interval entirely within the beneficial range, whereas the KD showed limited clinical relevance for FBG due to extremely high heterogeneity (I^2^ = 79.7%). For weight loss, the overall MD was −3.00 kg, but with substantially high heterogeneity (I^2^ = 93.9%) across subgroups. BMI outcomes were limited by extremely high heterogeneity (I^2^ = 97.5%) and all prediction intervals crossing zero, while waist circumference showed no statistically significant effect. None of the diets demonstrated a statistically significant advantage in lipid modification (TC, LDL-C, TG), with all confidence intervals crossing the line of no effect. Safety analyses showed KD was associated with a higher incidence of adverse events (gastrointestinal symptoms, transient hypoglycemia), while the other three diets exhibited favorable safety profiles.

**Conclusion:**

This meta-analysis descriptively supports the potential benefits of all four dietary patterns for glycemic control and weight reduction. However, due to substantial inter-study heterogeneity and prediction intervals crossing the line of no effect, the pooled estimates preclude definitive claims of relative superiority among diets. Consequently, personalized dietary strategies balancing efficacy, safety, and patient preferences are recommended, with cautious interpretation of indirect cross-diet comparisons.

**Systematic review registration:**

https://www.crd.york.ac.uk/PROSPERO/view/CRD420261478257, identifier PROSPERO (CRD420261478257).

## 1 Introduction

Type 2 diabetes mellitus (T2DM) is a prevalent chronic metabolic disease driven by insulin resistance and progressive pancreatic β-cell dysfunction. Globally, more than 500 million people are currently living with this condition (1–4). The continuously increasing prevalence of T2DM has created a heavy public health and economic burden worldwide, highlighting an urgent demand for accessible, effective and long-term management strategies. As a fundamental component of T2DM treatment, dietary intervention plays an irreplaceable role in clinical practice (5). Although anti-diabetic medications are indispensable for blood glucose regulation, rational dietary adjustment can effectively optimize glycemic control, lower cardiovascular disease risks and facilitate durable weight reduction, all of which are closely associated with the long-term prognosis of T2DM patients (6).

A wealth of studies has confirmed the therapeutic value of diverse dietary patterns for T2DM (7–11). Nevertheless, existing meta-analyses have notable shortcomings that restrict clinical application. First, published randomized controlled trials (RCTs) vary greatly in methodological quality. Common issues include incomplete anthropometric data, poor reporting of participant adherence and insufficient long-term follow-up. Second, most previous syntheses only focus on one single dietary regimen or head-to-head comparisons between two diets. Few studies have comprehensively evaluated multiple mainstream dietary approaches in parallel, covering their clinical efficacy, safety performance and underlying mechanisms such as gut microbiota regulation. Third, evidence concerning short-term intervention effects within approximately 3 months remains scarce. Such evidence is particularly valuable for patients who expect rapid metabolic improvement at the early stage of treatment (7). To address the above research gaps, the present systematic review and meta-analysis aims to synthesize available RCT evidence regarding four widely applied dietary patterns, namely the Mediterranean diet, intermittent fasting, energy-restricted diet and ketogenic diet. We specifically focus on intervention courses of 6 months, to provide targeted evidence for early metabolic management in T2DM patients. This work intends to generate comprehensive evidence to support individualized dietary strategies for T2DM care (12).

## 2 Materials and methods

This review was performed in accordance with a preregistered systematic review protocol (PROSPERO: CRD420261478257) and according to the Preferred Reporting Items for Systematic Reviews and Meta-analyses (PRISMA) (13). It should be noted that this study was retrospectively registered on PROSPERO. The original protocol planned to perform a network meta-analysis; however, due to insufficient network connectivity across outcomes, we were unable to conduct NMA and therefore employed pairwise meta-analyses instead. This protocol amendment was made prior to any formal analysis and was based on data availability constraints rather than on the analytic results.

### 2.1 Databases and retrieval strategies

The following databases were searched from inception to March 13, 2025, to identify studies: the Cochrane Library, Embase, PubMed and Web of Science. With the assistance of a professional clinical librarian, a retrieval strategy was customized, including the use of Cochrane-recommended filters to identify randomized controlled trials in PubMed. Retrieval Terms were as follows:(“Type 2 Diabetes Mellitus”[Mesh Terms] OR “Type 2 Diabetes”[Title/Abstract]) AND (“Diet”[Mesh Terms] OR “Mediterranean Diet”[Title/Abstract] OR “Ketogenic Diet”[Title/Abstract] OR “Intermittent Fasting”[Title/Abstract] OR “Energy-Restricted Diet”[Title/Abstract]) AND (“Randomized Controlled Trial”[pt]). Detailed search strategy is in Supplementary Table 1.

### 2.2 Inclusion and exclusion criteria

This review focused on four common dietary intervention protocols. The Mediterranean diet provides 50–60% of daily total calories from complex carbohydrates, 25–30% from monounsaturated and polyunsaturated fats, and 15–20% from protein. The ketogenic diet is defined by a daily carbohydrate intake of 20–50 g and a protein intake of 1 g per kilogram of body weight. The intermittent fasting regimen was defined as time-restricted eating (TRE) with a daily eating window of 6–10 h. The 5:2 diet or other intermittent fasting variants were not included in this definition. And the calorie-restricted diet was defined as a dietary approach that achieves caloric restriction by reducing total daily energy intake, either through a prescribed daily caloric target (e.g., 1,000–1,500 kcal/day) or through a daily reduction of approximately 500 kcal from baseline energy requirements. A unified short-term intervention window of 6 months was adopted to ensure the comparability of intervention outcomes and reduce confounding biases caused by declined dietary adherence and medication adjustments in long-term interventions. It should be noted that a proportion of the dietary intervention trials included in this review concurrently incorporated exercise or lifestyle intervention components. Therefore, the independent effect of dietary modification alone cannot be fully isolated from the pooled estimates. The combined effect sizes should thus be interpreted as the effects of diet-centered comprehensive lifestyle interventions, rather than pure dietary effects.

Studies were eligible for inclusion based on the following criteria: (1) original randomized controlled trials exploring the efficacy of the above four dietary patterns; (2) trials that set standard dietary patterns as the control group; (3) intervention duration within the range of 6 months; (4) trials permitting concurrent exercise, lifestyle and behavioral interventions; (5) no restrictions on publication language, publication date and publication status. Raw unpublished data from eligible published and unpublished trials were also collected to supplement the analysis.

Studies were excluded based on the following criteria: (1) animal experiments, non-randomized trials, observational studies, reviews, and case reports; (2) interventions with a duration shorter than 6 weeks; (3) studies focusing on gestational diabetes.

### 2.3 Outcome measures

This study evaluated outcomes across three domains. The first domain was glycemic control, which included fasting blood glucose and glycated hemoglobin (HbA1c). The second domain comprised anthropometric parameters, specifically body weight, body mass index (BMI), and waist circumference. The third domain was the lipid profile, including total cholesterol (TC), low-density lipoprotein cholesterol (LDL-C) and triglycerides (TG). Additionally, safety data were collected, including the type, incidence, and severity of adverse events.

### 2.4 Data extraction

After two researchers independently conducted database searches, duplicate studies were removed. Two independent reviewers first screened the title and abstract of each study to assess eligibility prior to full-text review of potentially qualified studies. Any discrepancies in the screening decisions for a specific study were resolved through discussion; if consensus failed to be reached, the opinion of a third reviewer was consulted. For crossover trials, only data from the first phase were extracted to maintain independence.

### 2.5 Risk of bias assessment

Data were independently extracted and processed from each clinical trial, including information on study design characteristics, study population, interventions, comparators, alternatives, and health outcomes. All outcomes were extracted and reported within the 6-month time point, consistent with the pre-specified intervention window. We assessed the risk of bias of included randomized trials via Cochrane ROB 2.0 ^[10]^ and graded each bias domain as high risk, low risk or some concerns with the official Excel template from the ROB development team. Publication bias test and sensitivity analysis were performed using R version 4.5.2.

### 2.6 Data analysis

Intervention outcomes were synthesized using the mean difference (MD) and standard deviation (SD) of pre- and post-intervention values. The I^2^ statistic was utilized to quantify statistical heterogeneity across the included studies. To better account for between-study heterogeneity and to estimate the expected range of true effects in future clinical settings, we calculated the 95% prediction intervals (PI) for all pooled estimates. Unlike the 95% confidence interval, which reflects the precision of the summary estimate, the prediction interval incorporates the between-study variance (τ^2^) and provides a range within which the true effect of a new study is likely to fall. Prediction intervals were computed using the standard formula under the random-effects model, assuming a t-distribution with k − 2 degrees of freedom, where k is the number of studies included in the meta-analysis. This approach was applied to all primary and secondary outcomes to facilitate a more cautious interpretation of the pooled results. Meta-regression with the metafor package in R 4.5.2 was conducted to identify heterogeneity sources and quantify the impacts of study-level covariates on intervention effects. Considering variations in ethnic backgrounds and baseline disease profiles among study populations, intervention effects were expected to differ across trials. Therefore, a random-effects model was adopted to pool weighted MD values. Sensitivity analysis was performed by sequentially omitting individual studies and excluding trials with a high risk of bias, to verify the robustness of the pooled results and further explore potential sources of heterogeneity. A two-tailed *p*-value < 0.05 was defined as statistically significant.

## 3 Results

### 3.1 Study characteristics

Our search yielded 6,759 records, of which 25 RCTs met the inclusion criteria. The trials varied in scale, with sample sizes ranging from 12 to 200 participants and mean participant ages spanning 37 to 67 years. Intervention durations ranged from 6 weeks to 6 months, with most trials concentrated around 12 weeks. The specific search process is shown in Figure 1. The intervention groups were assigned to four distinct dietary patterns. Detailed definitions and macronutrient compositions for each dietary intervention adhered to the inclusion criteria outlined in the Methods section. Control groups generally followed participants’ usual or standard diet for comparison. Detailed characteristics of the included studies are presented in Table 1.

**Figure 1:**
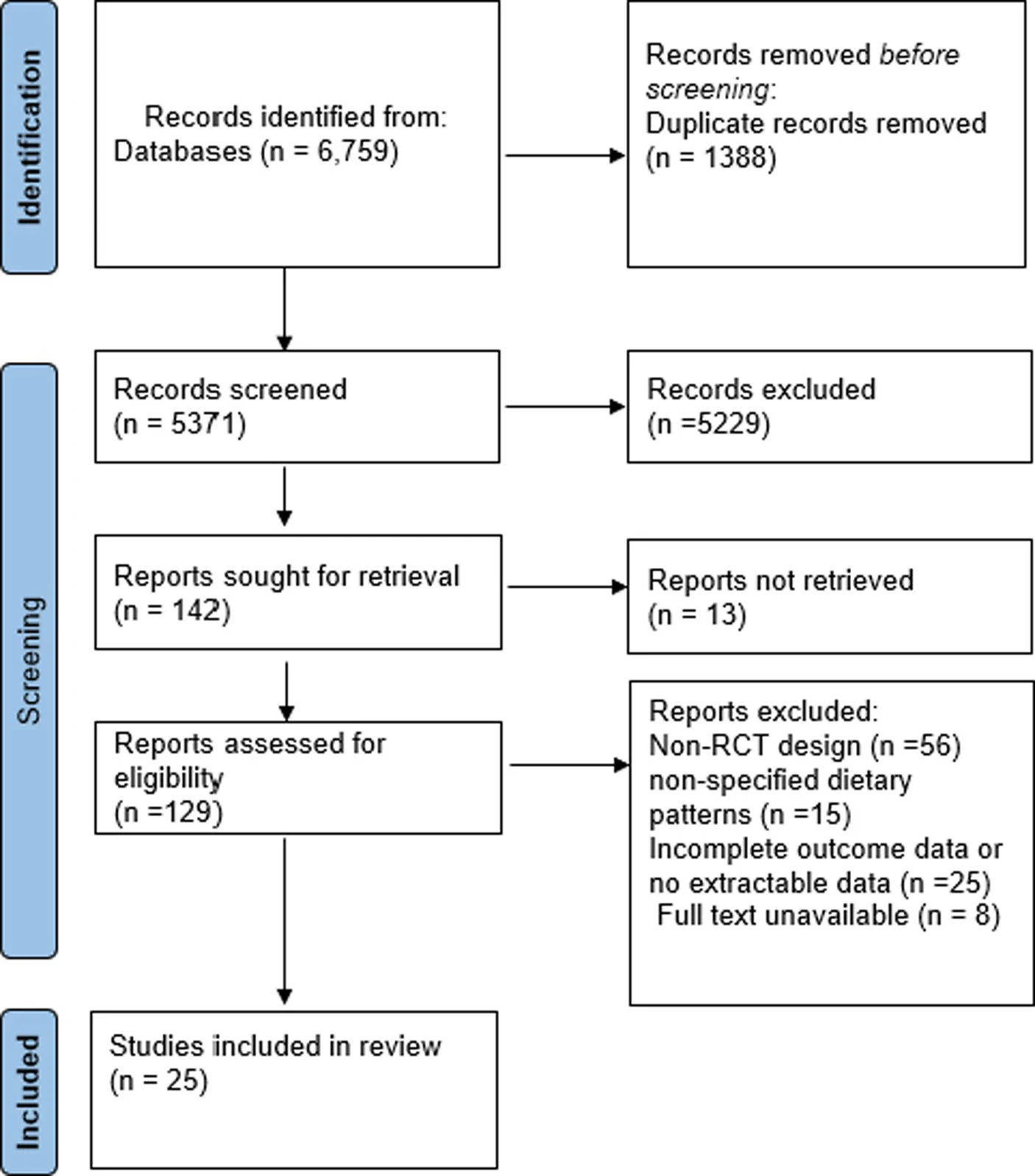
PRISMA flow diagram.

**Table 1:** Characteristic table.

Author (year)	Country	Participants	Sample size (T/C)	Age (Mean ± SD)	Intervention diet	Control diet
A. Fortin 2018	Canada	T2DM	14/14	50.9 ± 10.3	Use of olive oil as the main source of fat, multiple servings of fish per week and a limited intake of red and processed meat.	Similar diet, but with the following low-fat diet principles
Ana Arpón 2017	Spain	T2DM	12/12	62.3 ± 5.9	Standard Mediterranean diet	Daily diet
Domenico Tricò 2021	Italy	T2DM	15/17	44.2 ± 8.7	Rich in whole grains, eggs, poultry, fish, vegetables, legumes, fruits and olive oil as the main condiment, and low in red and processed meat	Made of beef, veal, cold cuts, eggs and seasoned cheese, vegetables and fruits
Jordi Salas-Salvad’o 2018	Spain	T2DM	327/299	64 ± 5	Standard Mediterranean diet	Daily diet
Katherine Esposito 2009	USA	T2DM	108/107	52 ± 10	Rich in vegetables and whole grains, low in red meat (replaced by poultry and fish), with energy restricted to 1,500 kcal/day for women and 1800 kcal/day for men.	Rich in whole grains and restricted additional fats, sweets, and high-fat snacks
Rosario Alonso-Domínguez 2019	Spain	T2DM	102/102	60 ± 7	Olive oil as the main fat for cooking	Daily diet
Xavier Pintó 2019	Spain	T2DM	34/30	64 ± 5	The Mediterranean diet is rich in extra virgin olive oil	Daily diet
Charlotte Andriessen 2022	Netherlands	T2DM	14/13	63 ± 6	Consume their habitual diet within a 10 h window during the daytime, with the last meal completed no later than 18:00 h	Unlimited
Fang Tang 2020	China	T2DM	22/24	50 ± 8	Consume their habitual diet within a 10 h window during the daytime, with the last meal completed no later than 18:00 h	Unlimited
S. Carter 2019	Australia	T2DM	70/67	61 ± 9	Consume their habitual diet within a 10 h window during the daytime, with the last meal completed no later than 18:00 h	Unlimited
A Flood 2008	USA	T2DM	248/502	61 ± 5	Low-fat (20% of energy), high-fiber (18 g per 1,000 kcals of energy intake) and high-fruit and -vegetable (5–8 servings per day) diet	Unlimited
Bettina Nowotny 2014	USA	T2DM	20/17	55 ± 7	Daily diet sheets providing a 1,256-kJ reduction in total daily energy intake and a constant macronutrient distribution	Unlimited
Josée Leroux-Stewart 2020	Canada	T2DM	21/16	62 ± 6	At least 1,200 Kcal/day	Unlimited
Vasiliki Argiana 2014	Greece	T2DM	28/30	61 ± 5	Reduce your daily diet by 500 calories	Unlimited
Martí Juanola-Falgarona 2014		T2DM	40/40	47 ± 5	Reduce your daily diet by 500 calories	Unlimited
Piero Ruggenenti MD 2016	USA	T2DM	34/36	60 ± 7	Provide 45–50% of energy from carbs, 30–35% from fat, 15–20% from proteins, 100% of RDI for micronutrients, >20 g/d fiber, and <300 mg/d cholesterol.	Unlimited
Salwa W. Rizkalla, MD, PHD 2004	France	T2DM	12/12	54 ± 2	Reduce your daily diet by 500 calories	Unlimited
Thomas PJ Solomon 2010	USA	T2DM	10/12	67 ± 5	Received a diet (55% carbohydrate, 30% fat, and 15% protein	Unlimited
Xiao Yang 2021	China	T2DM	36/36	53 ± 5	840 kcal/day	Unlimited
A Goday 2016	Spain	T2DM	45/44	54 ± 5	A very low-calorie diet (600–800 kcal per day), low in carbohydrates (50 g daily from vegetables) and lipids (only 10 g of olive oil per day)	Unlimited
Eva M. Gram-Kampmann MD 2022	Denmark	T2DM	49/22	57 ± 5	A maximum of 20 E% of carbohydrates (mainly complex and water-soluble), 50–60 E% fat, and 25–30 E% protein	Consisting of 50–60 E% carbohydrates mainly from fruit, vegetables, and whole-grain sources, 20–30 E% fat.
Jeannie Tay PhD 2012	Australia	T2DM	58/57	58 ± 6	LC diet: 14% CHO (<50 g/d), 28% pro, 58% fat (35% MUFA, 13% PUFA)	Unlimited
Laura R. Saslow 2017	USA	T2DM	18/16	54 ± 5	Reduce their carbohydrate intake to between 20–50 g of carbohydrates (excluding fiber) a day	Unlimited
Nur Aisyah Zainordin 2021	Malaysia	T2DM	14/16	56 ± 5	Reduce your daily diet by 500 calories	Unlimited
Sumei Li 2022	China	T2DM	24/29	36 ± 13	Included olive oil, butter, pan - fried salmon, Pacific saury, sardines, broccoli, avocado, etc. daily ingredient limits: 30–50 g carbs, 60 g protein, 130 g fat, (1,500 ± 50) Kcal total calories.	Foods unrestricted. Daily ingredient limits: 250–280 g carbs, 60 g protein, 20 g fat, (1,500 ± 50) Kcal total calories.

### 3.2 Study quality and risk of bias assessment

The summary of the bias assessment risks included in the study is shown in Figure 2. The risk of bias assessment results for each included study are presented in Supplementary Figure 1. The overall methodological quality of the included literature was satisfactory; only a small number of studies exhibited potential bias concerns, while the vast majority were categorized as having a low risk of bias. Publication bias was assessed using funnel plots, supplemented by Egger’s test and Begg’s test (Supplementary Figures 2–9). The results showed that neither Egger’s test (*p* = 0.5427) nor Begg’s test (*p* = 0.4264) indicated significant publication bias for TC. However, Egger’s test *p*-values were less than 0.05 for Weight (*p* = 0.0165), BMI (*p* = 0.0177), Fasting blood glucose (*p* = 0.0305), and TG (*p* = 0.0467), suggesting possible small-study effects or publication bias. For these outcomes, Begg’s test did not reach statistical significance (all *p* > 0.05). Considering the differences in statistical power between Egger’s and Begg’s tests and the limited number of studies in some subgroups, these findings should be interpreted with caution. For waist circumference, only four studies were included, and statistical tests were of limited reference value; therefore, only visual inspection of the funnel plot was performed. Leave-one-out analysis showed the positive findings for weight, BMI, glycemia, and HbA1c were stable and not driven by a single study (Supplementary Figures 10–17). However, overall heterogeneity was high, and prediction intervals crossed the null, limiting clinical predictive value. Conversely, LDL-C, TC, TG, and waist circumference consistently showed no benefit or were highly unstable.

**Figure 2:**
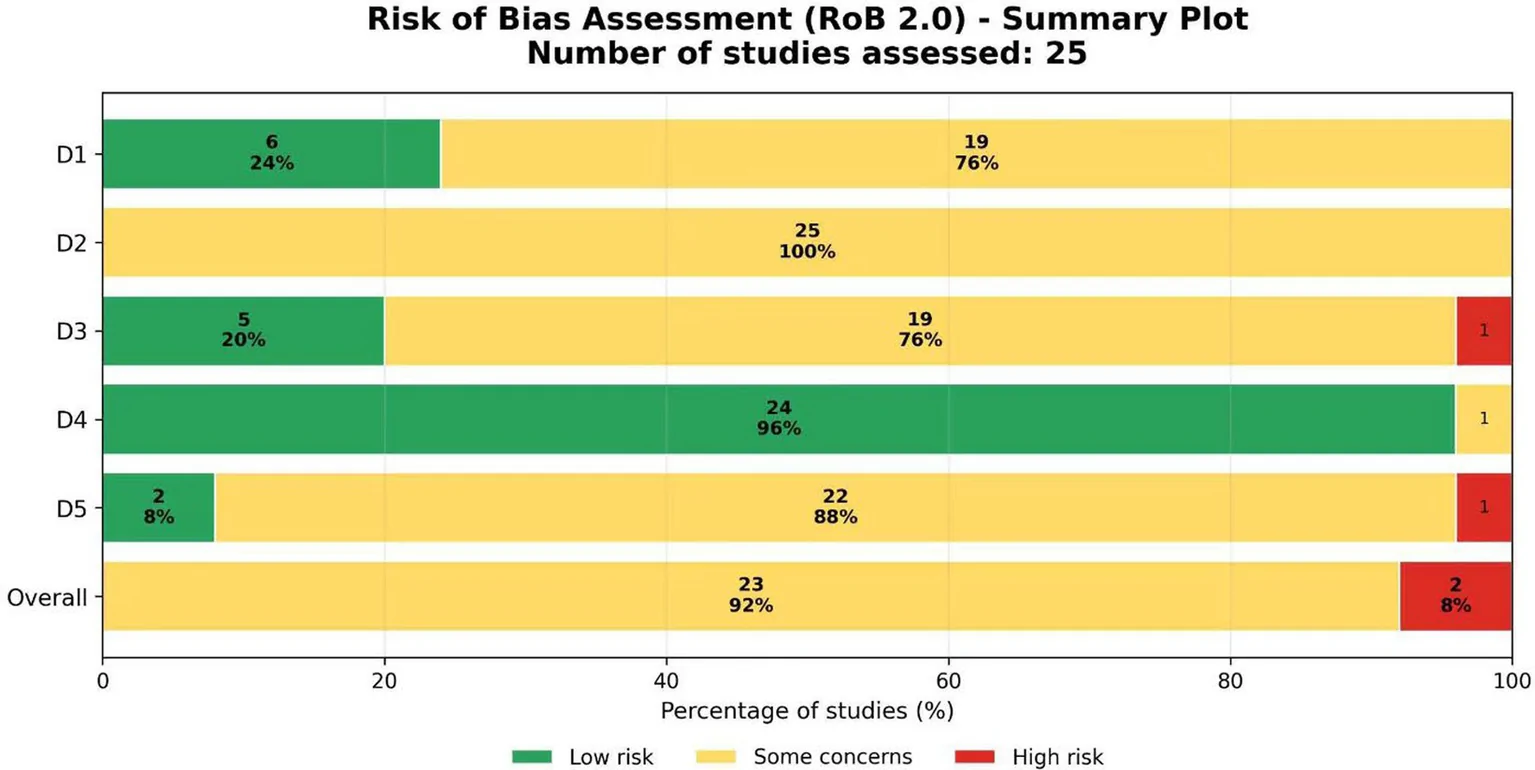
Summary plot.

### 3.3 Glycemic indicators

#### 3.3.1 Fasting blood glucose

Overall, the pooled results indicated that all four dietary patterns reduced fasting blood glucose (MD = −0.63), with moderate heterogeneity (I^2^ = 62.1%). In the subgroup analysis, the pooled effect estimate for the ketogenic diet (MD = −0.90) demonstrated limited clinical relevance, attributable to its extremely high heterogeneity (I^2^ = 79.7%) and a 95% prediction interval that crossed the null line ([−2.23; 0.43]). In contrast, although the Mediterranean diet yielded a smaller effect size (MD = −0.40), it exhibited low heterogeneity, and its prediction interval ([−0.59; −0.20]) fell entirely within the beneficial range (excluding zero). This suggests a high probability of achieving stable glucose-lowering effects in future clinical applications, making it the most robust dietary intervention. Figure 3 is a forest diagram of fasting blood glucose.

**Figure 3:**
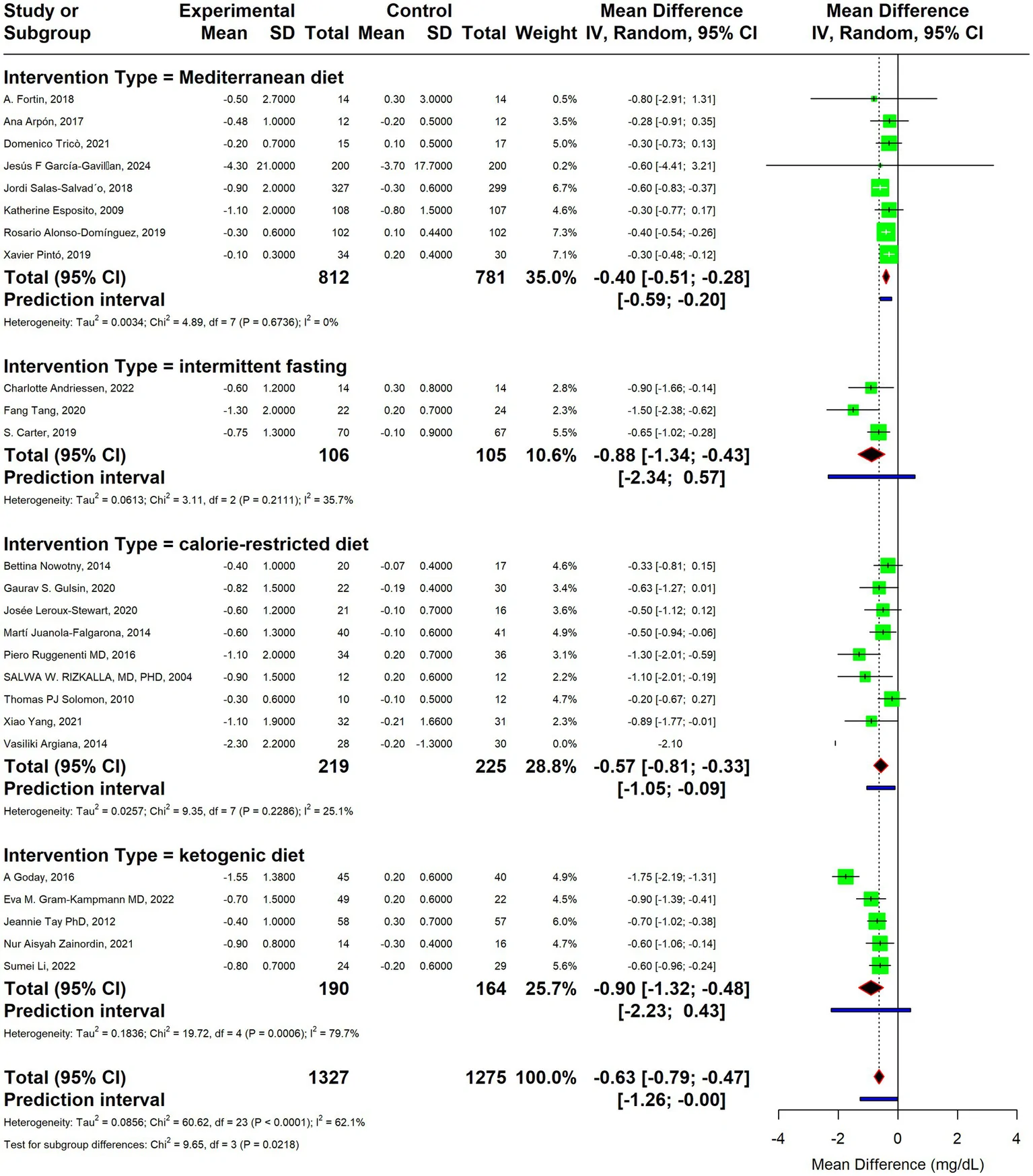
Forest plot of fasting blood glucose.

#### 3.3.2 Glycated hemoglobin

The pooled analysis (MD = −0.50 percentage points; 95% CI: −0.65 to −0.36 percentage points) was limited by substantial heterogeneity (I^2^ = 83.8%) and a prediction interval crossing zero (PI: −1.04 to 0.03), rendering the overall estimate clinically unreliable. In subgroup analyses, the calorie-restricted (MD = −0.61 percentage points; I^2^ = 15.5%) and ketogenic diets (MD = −0.46 percentage points; I^2^ = 14.8%) demonstrated robust and consistent effects, with prediction intervals entirely within the beneficial range. Conversely, the Mediterranean diet and intermittent fasting had high heterogeneity and prediction intervals crossing the null line, indicating unstable effects. Thus, calorie-restricted and ketogenic diets are more reliable strategies; however, this pairwise meta-analysis cannot establish relative superiority between diets. Figure 4 is a forest diagram of Glycated hemoglobin.

**Figure 4:**
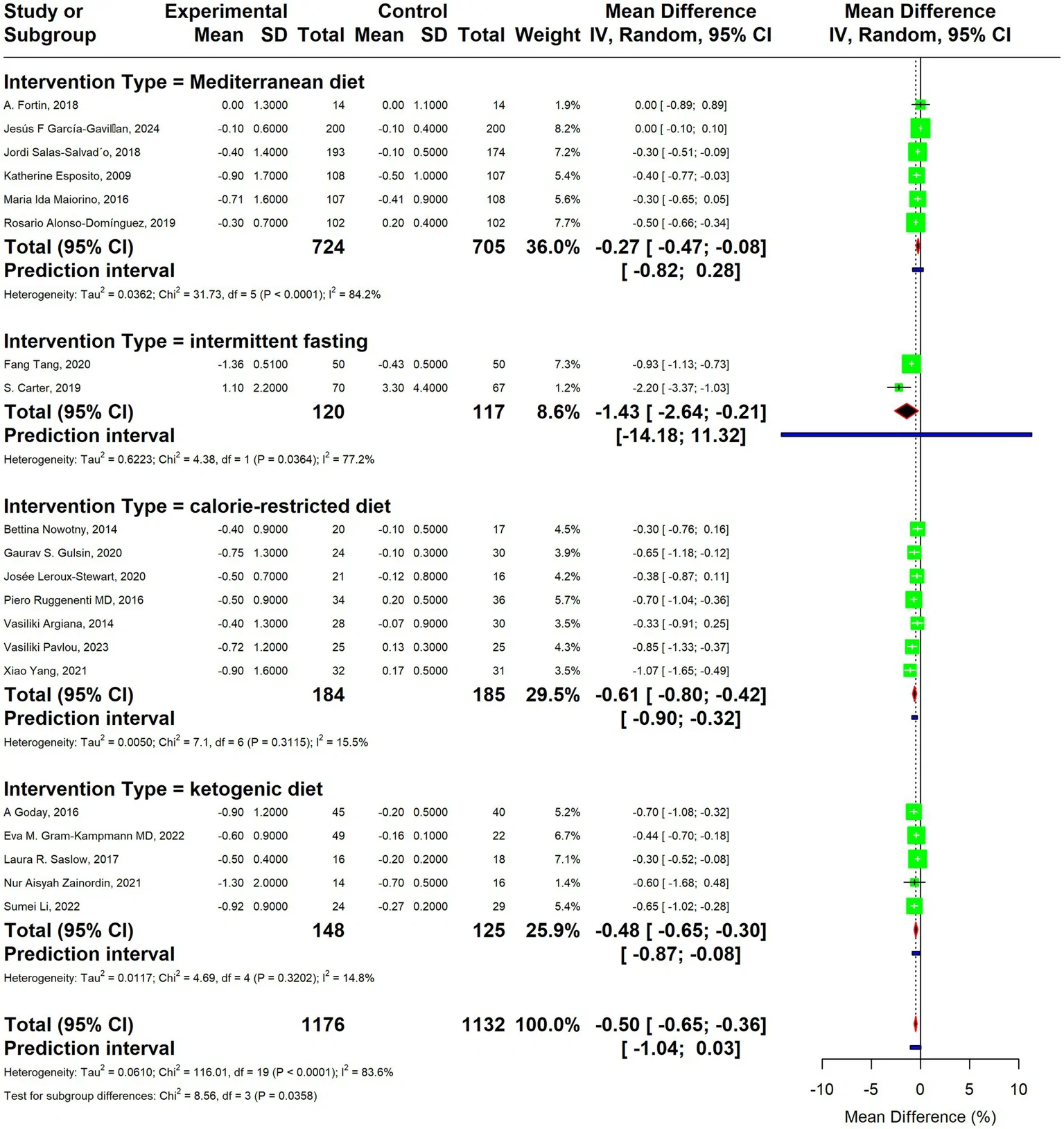
Forest plot of glycated hemoglobin.

#### 3.3.3 Meta-regression analysis

To explore the potential sources of heterogeneity observed across the metabolic indicators, a meta-regression analysis was performed with publication year and average sample size as covariates. The regression plot for publication year (Left) shows a relatively flat slope, indicating that the effect sizes (SMD) have remained consistent over time. Conversely, the analysis of average sample size (Right) reveals an upward trend, suggesting that studies with larger sample sizes tended to report smaller effect sizes (SMD closer to zero), whereas smaller studies demonstrated larger reductions. Statistical analysis indicated that neither covariate significantly moderated the treatment effects (*P* > 0.05), implying that the observed heterogeneity is likely driven by other unmeasured factors, such as dietary adherence. Supplementary Figure 18 is a meta-regression analysis of blood glucose indicators.

### 3.4 Anthropometric indices

#### 3.4.1 Weight

In these studies assessing weight loss (*n* = 2,159), the overall pooled MD was −3.00 kg (95% CI: −4.31 to −1.69; I^2^ = 93.9%). Subgroup analyses showed that the MD for the Mediterranean diet was −1.44 kg (95% CI: −2.40 to −0.48; I^2^ = 88.6%), for intermittent fasting was −0.66 kg (95% CI: −0.87 to −0.45; I^2^ = 0%), for the energy-restricted diet was −3.35 kg (95% CI: −4.81 to −1.89; I^2^ = 89.9%), and for the ketogenic diet was −4.87 kg (95% CI: −8.35 to −1.40; I^2^ = 92.3%). Subgroup differences were statistically significant (*p* < 0.001). However, given the substantial heterogeneity observed in most subgroups, these results should be interpreted descriptively. Figure 5 is a forest diagram of Weight.

**Figure 5:**
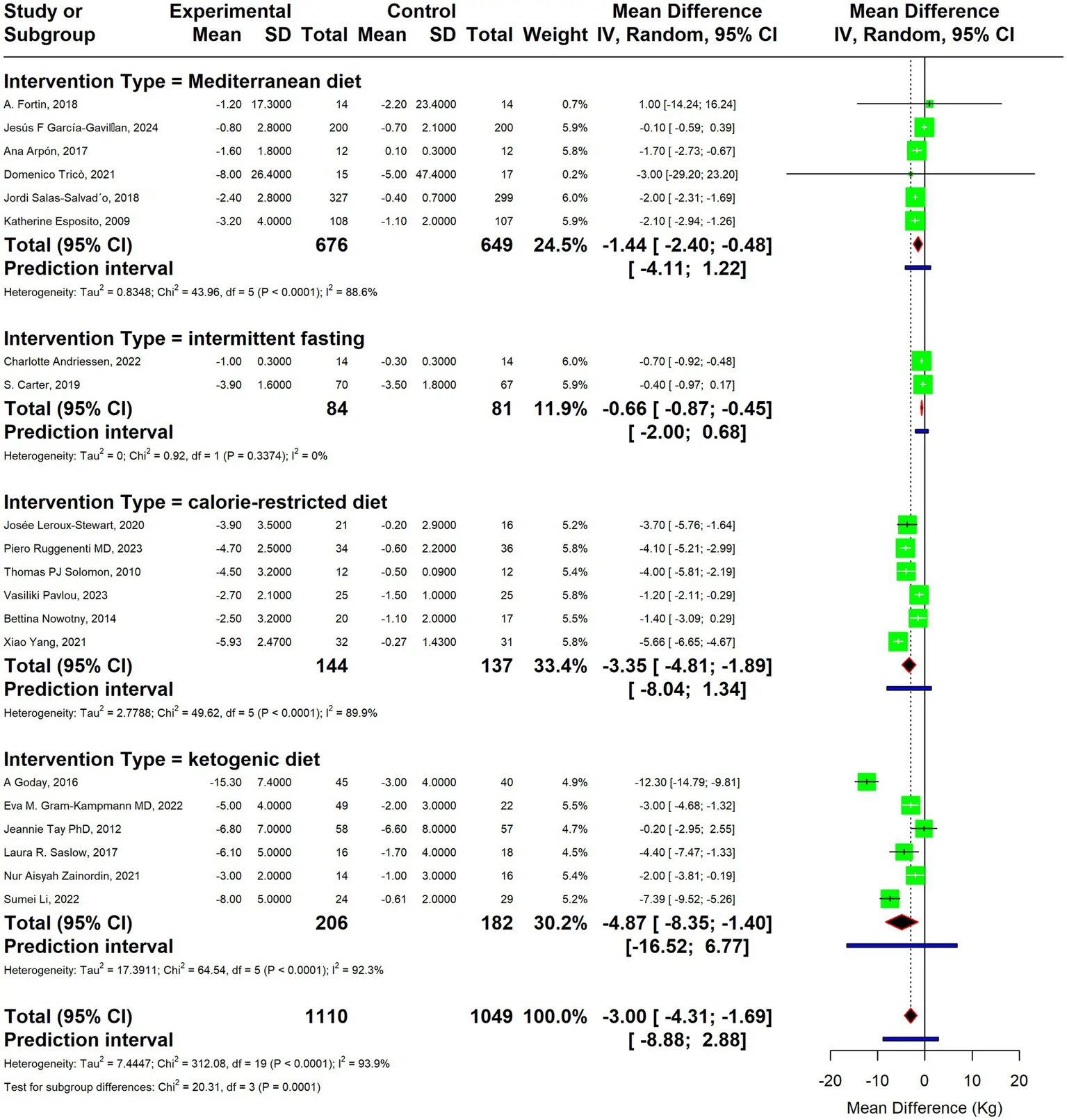
Forest plot of weight.

#### 3.4.2 BMI

The overall pooled mean difference for BMI was −1.73 kg/m^2^ (95% CI: −2.44 to −1.03; I^2^ = 97.5%; *p* < 0.00001). Subgroup analyses showed MDs of −0.62 (95% CI: −0.89 to −0.34; I^2^ = 88.3%) for the Mediterranean diet, −1.69 (95% CI: −4.80 to 1.43; I^2^ = 89.5%) for intermittent fasting (non-significant), −2.95 (95% CI: −4.49 to −1.42; I^2^ = 92.4%) for the calorie-restricted diet, and −1.78 (95% CI: −2.90 to −0.67; I^2^ = 97.5%) for the ketogenic diet. Subgroup differences were statistically significant (*p* = 0.0059). However, due to the extremely high heterogeneity (I^2^ > 80%) and the fact that the 95% prediction intervals for all subgroups and the overall analysis crossed the line of no effect, these findings should be interpreted descriptively without inferring relative superiority among diets. Figure 6 is a forest diagram of BMI.

**Figure 6:**
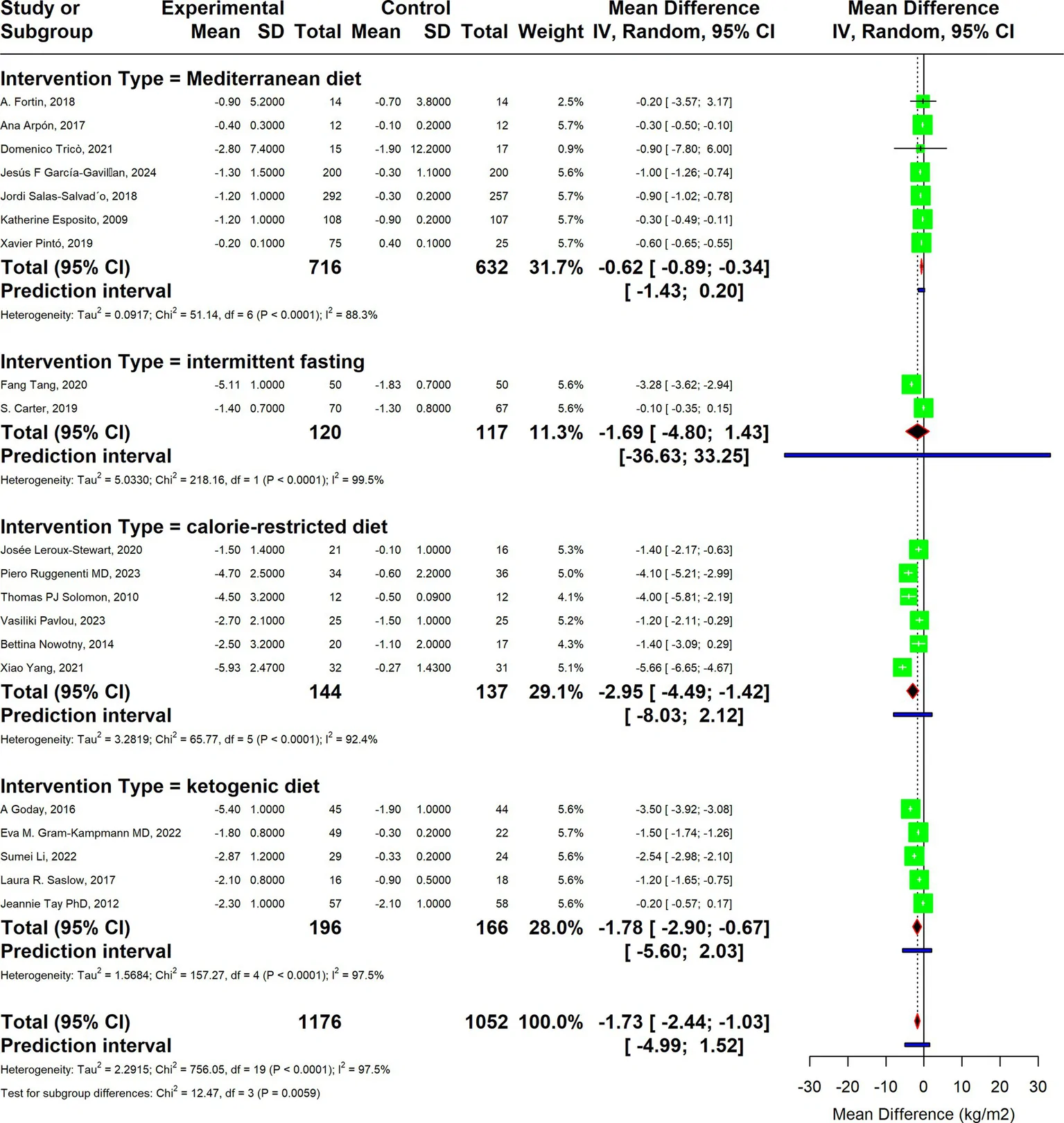
Forest plot of BMI.

#### 3.4.3 Waist circumference

The analysis included 4 studies (*n* = 675). The pooled mean difference (MD) was −1.96 cm (95% CI: −5.15 to 1.23; I^2^ = 93.7%). As the 95% confidence interval crossed the line of no effect, the pooled result was not statistically significant. The 95% prediction interval (−11.32 to 7.40) also crossed zero, indicating substantial variability. Figure 7 is a forest diagram of Waist circumference.

**Figure 7:**
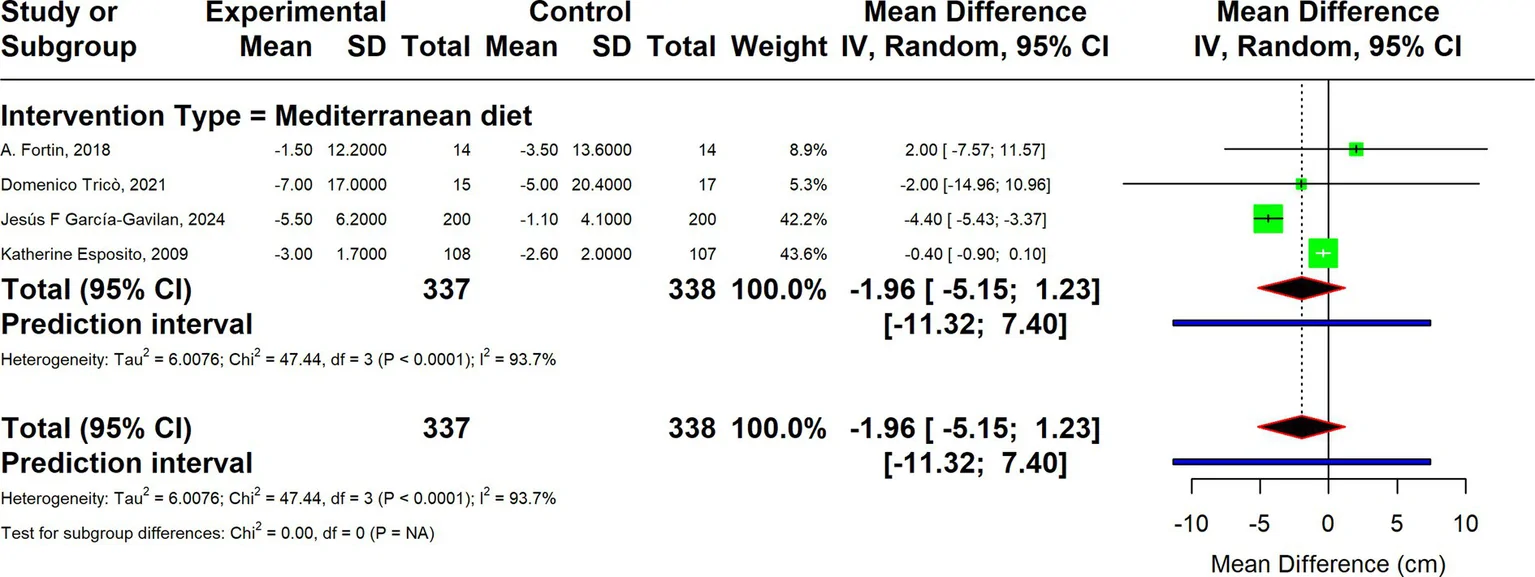
Forest plot of waist circumference.

#### 3.4.4 Meta-regression analysis

Meta-regression explored the high heterogeneity (I^2^ > 90%) in anthropometric outcomes using publication year and sample size as covariates. Publication year showed a significant negative association with SMD (slope = −0.08, *p* = 0.03), indicating that recent studies reported larger reductions. Conversely, sample size exhibited a positive trend (slope = 0.04, *p* = 0.02), suggesting that larger studies yielded more conservative effect sizes compared to smaller trials. These findings identify both factors as significant sources of the observed heterogeneity. Supplementary Figure 19 is a meta-regression analysis of Anthropometric Indices.

### 3.5 Lipid profile

#### 3.5.1 Low-density lipoprotein cholesterol

The analysis included 22 studies (*n* = 2,320). The overall pooled mean difference (MD) for LDL-C was −0.10 mmol/L (95% CI: −0.23 to 0.03; I^2^ = 97.1%). Since the 95% confidence interval crossed the line of no effect, the overall pooled result was not statistically significant. Subgroup analyses showed MDs of −0.09 (95% CI: −0.16 to −0.02) for the Mediterranean diet (*n* = 7), −0.77 (95% CI: −1.75 to 0.21) for intermittent fasting (*n* = 2), −0.02 (95% CI: −0.08 to 0.05) for the energy-restricted diet (*n* = 8), and −0.01 (95% CI: −0.24 to 0.22) for the ketogenic diet (*n* = 6). Subgroup differences were not statistically significant (*p* = 0.208). Figure 8 is a forest diagram of Low-Density Lipoprotein Cholesterol.

**Figure 8:**
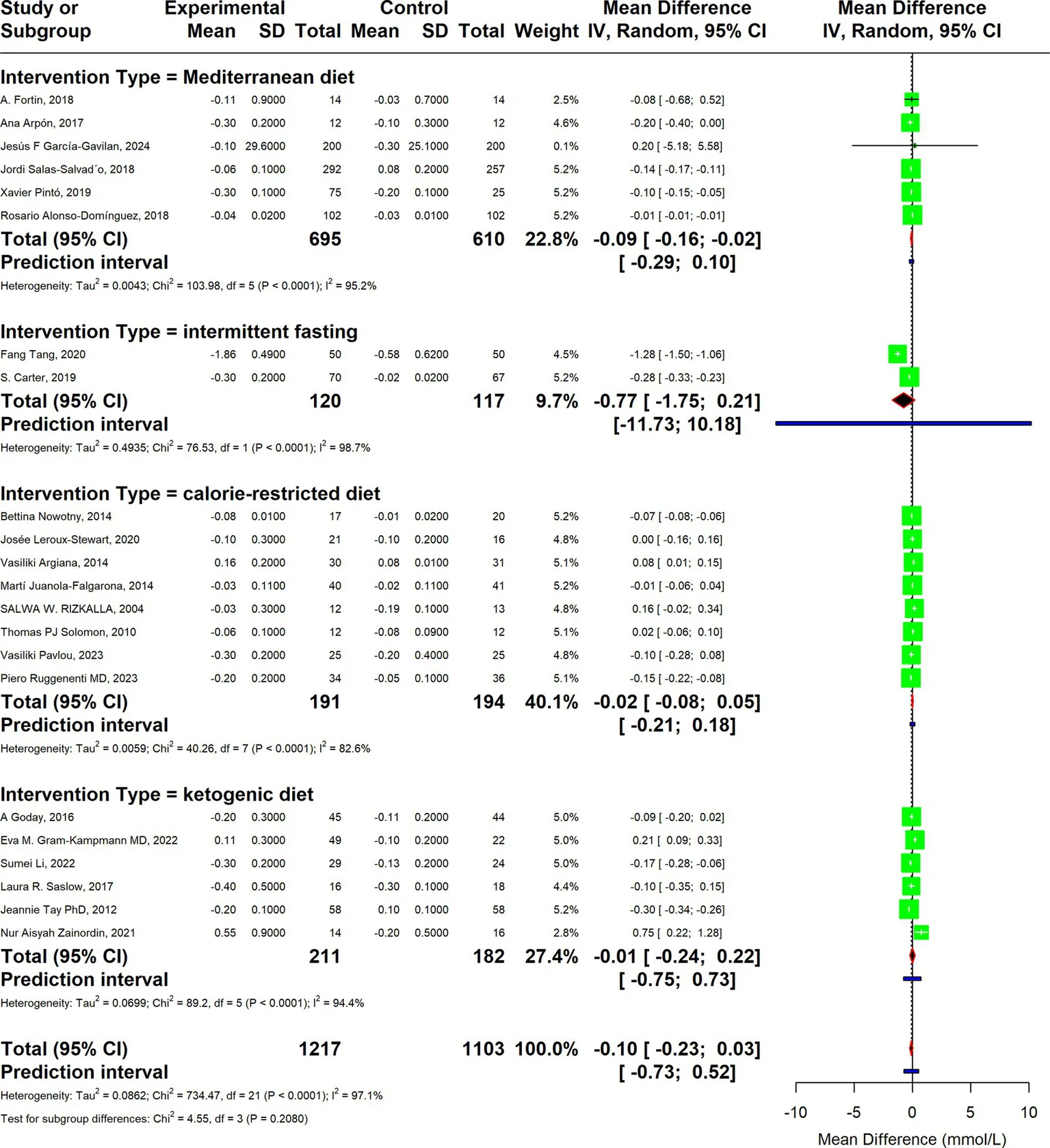
Forest plot of low-density lipoprotein cholesterol.

#### 3.5.2 Total cholesterol

Analyses showed MDs of 0.26 (95% CI: −0.11 to 0.63; I^2^ = 97.7%) for the Mediterranean diet (*n* = 6), 0.11 (95% CI: −0.21 to 0.42; I^2^ = 98.8%) for intermittent fasting (*n* = 2), −0.06 (95% CI: −0.67 to 0.54; I^2^ = 98%) for the energy-restricted diet (*n* = 6), and −0.02 (95% CI: −0.44 to 0.39; I^2^ = 97.5%) for the ketogenic diet (*n* = 3). All confidence intervals crossed the line of no effect, and no significant subgroup differences were observed (*p* = 0.7184). Furthermore, all prediction intervals crossed zero (e.g., overall PI: −1.16 to 1.28), and heterogeneity was extremely high (I^2^ > 97%). Therefore, these findings should be interpreted descriptively, indicating that no statistically significant effect on total cholesterol was observed for any dietary pattern in this analysis. Figure 9 is a forest diagram of Total Cholesterol.

**Figure 9:**
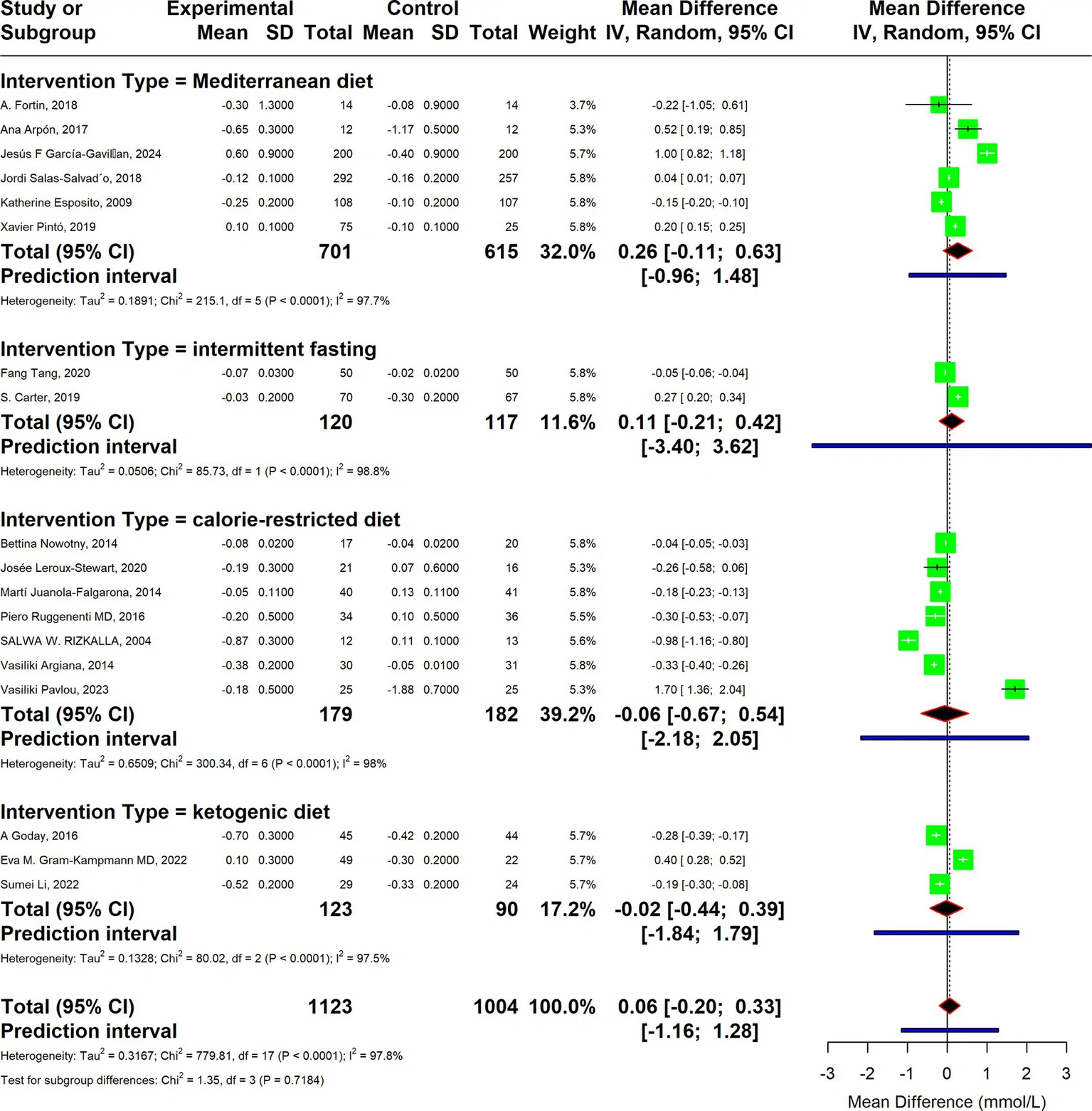
Forest plot of total cholesterol.

#### 3.5.3 Triglycerides

Across all four dietary patterns, the 95% prediction intervals crossed the line of no effect, and the I^2^ values exceeded 79% (and were above 94% for most). The test for subgroup differences was not statistically significant (*p* = 0.4876). This indicates substantial individual variability and internal inconsistency in the TG-lowering effects across the dietary patterns. Therefore, these findings do not provide sufficient evidence to demonstrate the superiority of any single dietary pattern over the others in reducing TG. Figure 10 is a forest diagram of Triglycerides.

**Figure 10:**
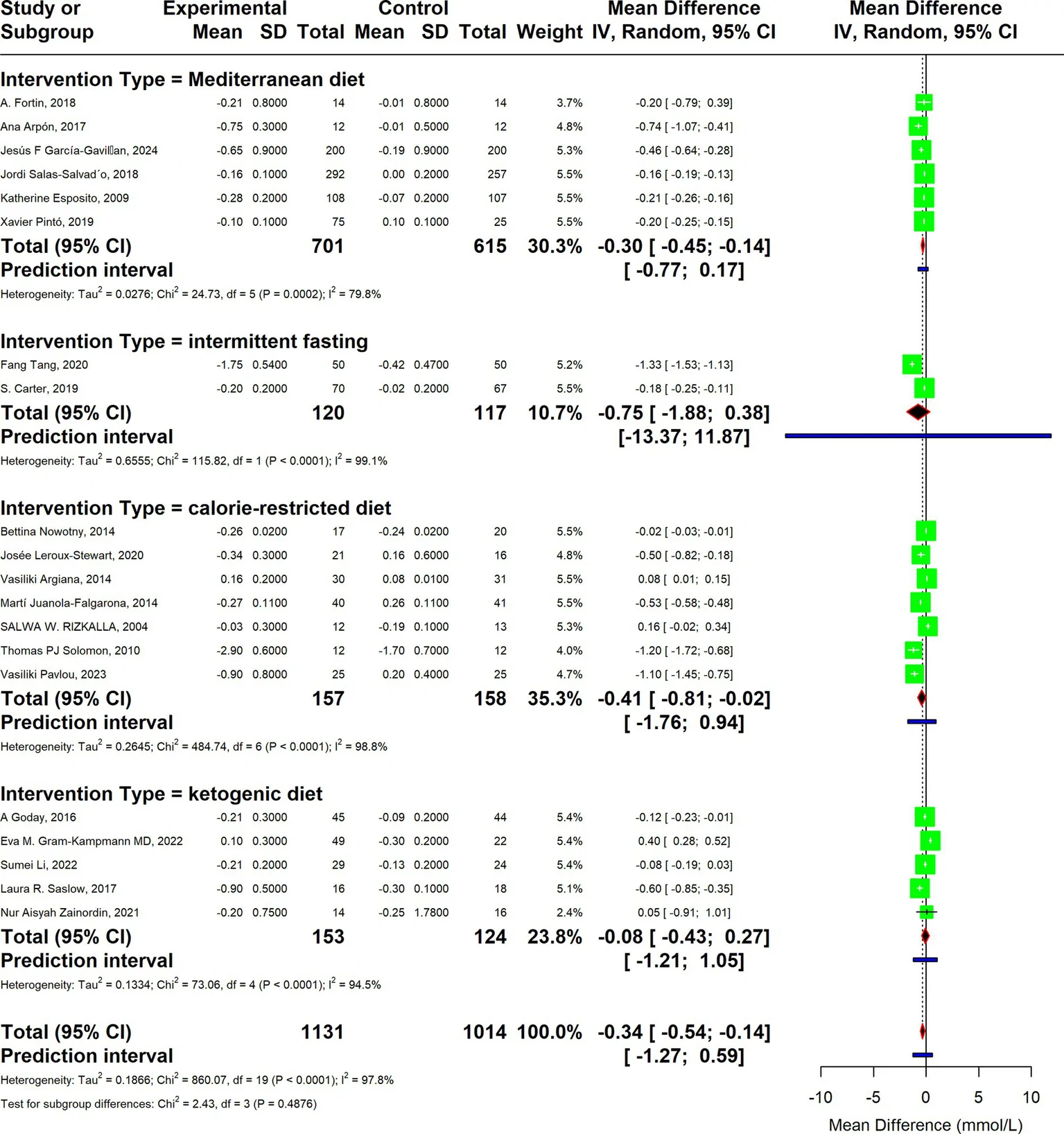
Forest plot of triglycerides.

#### 3.5.4 Meta-regression analysis

Given the extreme heterogeneity (I^2^ > 90%) observed across all lipid indices, a meta-regression analysis was performed to identify potential moderators. The regression plots (representative data shown) indicate that publication year (Left) had a minimal impact on the effect sizes, showing a relatively flat slope. However, average sample size (Right) displayed a notable upward trend. Since the primary outcomes (MD) are negative for most lipid reductions, this upward slope suggests that studies with larger sample sizes tended to report effect sizes closer to zero (more conservative results), whereas smaller studies were associated with larger magnitude reductions. This “small-study effect” may partially explain the observed high heterogeneity in the lipid data. Supplementary Figure 20 is a meta-regression analysis of Lipid Profile.

### 3.6 The safety of four types of diet

Safety data were extracted from a subset of included RCTs. Mediterranean diet, intermittent fasting and energy-restricted diet exhibited favorable safety profiles with minimal adverse events. Only mild transient hypoglycemia (1.3% of participants) and slight hunger discomfort were occasionally reported, none requiring clinical management or trial withdrawal. By comparison, KD and very low-calorie ketogenic (VLCK) interventions were associated with a higher rate of adverse symptoms, which mostly emerged in the initial dietary adaptation phase, widely referred to as “keto flu.” Key safety outcomes are summarized below: Gastrointestinal and systemic symptoms: In a VLCK trial (*n* = 45), prevalent adverse reactions included headache (20%), nausea (20%), constipation (17.8%), asthenia (15.6%), and vomiting (15.6%), while orthostatic hypotension occurred in 13.3% of participants. Another low-carbohydrate study reported constipation (10.2%) and abdominal discomfort (6.1%) in the intervention group, whereas no such symptoms were observed in controls.

Hypoglycemia: Ketogenic regimens were linked to more frequent hypoglycemic episodes, though these events were generally mild and self-limiting. Symptomatic hypoglycemia primarily arose within the first 4 weeks of intervention (10 versus 2 episodes in controls) and resolved following dietary adaptation. Biochemical hypoglycemia (<3.9 mmol/L) was uncommon, with an incidence of 0.067%.

Other adverse reactions: Mild hair loss (4.4%) and muscle cramps were occasionally reported under strict ketogenic diets. It is noteworthy that the musculoskeletal discomfort (approximately 18%) observed in the low-carbohydrate trials was found within the concurrent exercise intervention groups. As such, this outcome cannot be causally attributed to the dietary intervention alone, highlighting the necessity for further research to elucidate the contributing factors.

Renal safety: No severe adverse events concerning renal impairment were documented across the included studies. The data is shown in Figure 11.

**Figure 11:**
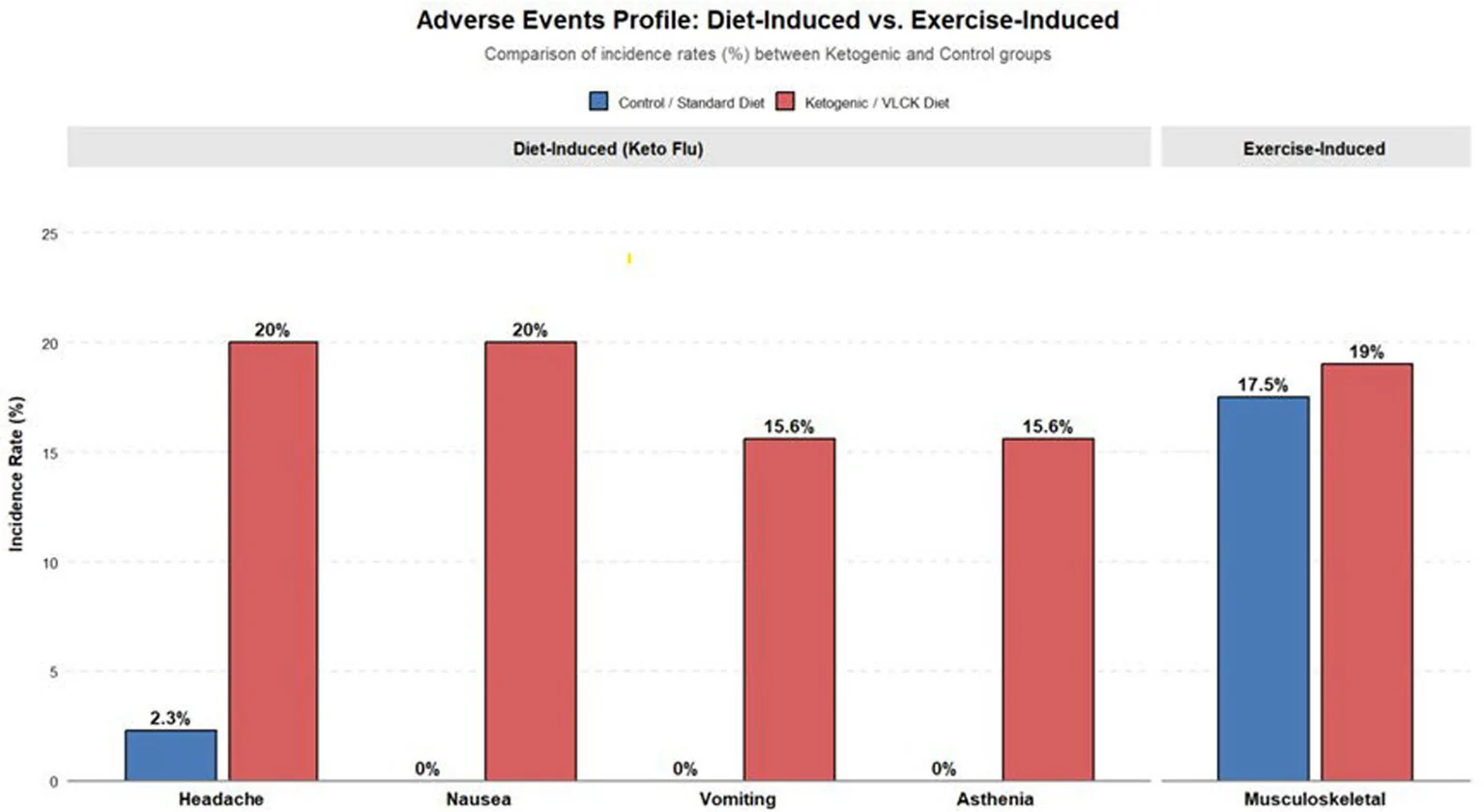
Safety summary.

## 4 Discussion

This systematic review and meta-analysis of randomized controlled trials comprehensively evaluated the effects of four common dietary patterns, including MD (14), IF (15), ERD (16), and KD (17), on glycemic regulation, weight modification, and cardiometabolic profiles in patients with T2DM. This study addresses key methodological shortcomings and insufficient comprehensive safety evidence observed in previous comparative dietary studies (18). Across all four dietary strategies, reductions in FBG and HbA1c were observed, although the pooled estimates were limited by substantial heterogeneity and prediction intervals crossing the line of no effect. Notably, the pooled estimate for the ketogenic diet on FBG showed limited clinical relevance, attributable to extremely high heterogeneity (I^2^ = 79.7%), whereas it showed lower heterogeneity for HbA1c (I^2^ = 14.8%). The Mediterranean diet yielded smaller effect sizes but exhibited excellent cross-study consistency, with its prediction interval entirely within the beneficial range. This consistency may be associated with enhanced insulin sensitivity and gut microbiota modulation (19, 20). The energy-restricted diet also improved glycemic control, possibly by inducing caloric deficit to alleviate obesity-related insulin resistance (21–23); moderate heterogeneity may reflect variations in intervention intensity across trials.

In terms of anthropometric outcomes, greater numerical reductions in body weight were observed for ERD and KD, though high heterogeneity limited the generalizability of these findings. MD was associated with a reduction in BMI; however, the pooled result for waist circumference was not statistically significant, with a confidence interval crossing zero. Overall, the lipid outcomes (TC, LDL-C, and TG) did not show statistically significant differences across the four dietary patterns, and subgroup differences were not significant, indicating that none of the diets demonstrated a clear advantage in lipid modification.

Significant heterogeneity was detected across primary outcomes, particularly for body weight, BMI, and LDL-C, which reflects the inherent variability of dietary interventions in T2DM management (24–26). Predefined meta-regression analyses provided partial explanations. For weight-related outcomes, publication year showed a negative correlation with effect size (indicating that recent studies tended to report larger reductions), whereas sample size showed a positive correlation (indicating that larger studies yielded more conservative effect sizes). Similarly, publication year and sample size significantly moderated lipid-related outcomes. However, neither factor altered FBG outcomes, implying that unmeasured variables, such as dietary adherence and concurrent physical activity, may primarily account for the residual heterogeneity. Heterogeneity in BMI and WC may also stem from inconsistent intervention intensity and variable baseline fat distribution. These findings underscore the complex, multifactorial nature of dietary interventions and highlight the necessity of cautious interpretation and personalized dietary prescription for T2DM (27, 28). Future studies should adopt standardized intervention protocols, detailed adherence documentation, and stratified study designs to reduce methodological diversity and improve result generalizability.

Safety analyses demonstrated a distinct adverse event profile for KD, which requires close clinical supervision due to higher rates of gastrointestinal and metabolic side effects, whereas the other three dietary patterns were generally well-tolerated with fewer reported adverse events (29, 30). Subgroup microbiota analyses indicated that dietary interventions, particularly high-fiber MD regimens (31, 32), improve microbial diversity and short-chain fatty acid production, although the direct causal linkage between microbial alterations and metabolic benefits remains unclear (33).

The present analysis has several limitations. First, substantial inter-study heterogeneity, relatively short intervention durations (ranging from 6 weeks to 6 months, with most trials concentrated around 12 weeks), and insufficient long-term outcome data limit the generalizability of our findings. Furthermore, a proportion of included trials bundled dietary intervention with exercise or lifestyle counseling, which may inflate the apparent independent effect of diet alone and increase heterogeneity. Therefore, the pooled effects should be interpreted as the overall effect of diet-centered comprehensive lifestyle interventions in real-world settings, rather than the pure dietary effect per se. Future studies should design standardized protocols to isolate the contributions of diet and exercise and explore dose–response relationships across varying intervention durations (34). The findings for intermittent fasting in this study are primarily applicable to time-restricted eating (TRE) protocols, as all included trials were TRE types; the results should not be extrapolated to 5:2 diets or other IF variants.

Moreover, we acknowledge that the control conditions (e.g., caloric intake, macronutrient composition, intensity of guidance) varied across dietary groups. Therefore, cross-diet indirect comparisons based on the current pairwise meta-analysis are unreliable, and readers should interpret these results with caution. To truly address the relative superiority of dietary patterns, future well-designed randomized controlled trials (RCTs) with direct head-to-head comparisons across different diets are warranted, providing a robust data foundation for network meta-analyses (NMA). Additionally, it should be acknowledged that this study was retrospectively registered on PROSPERO, and the analysis plan was amended from a network meta-analysis to pairwise meta-analyses due to insufficient network connectivity across outcomes. This amendment was made based on data availability constraints prior to formal analysis.

In conclusion, with appropriate caution and personalized consideration, this meta-analysis descriptively supports the potential of these dietary patterns for T2DM management: the Mediterranean diet shows stable and low-risk glycemic control; the energy-restricted diet shows benefits in weight reduction; evidence regarding visceral fat reduction for intermittent fasting is inconclusive due to limited studies; and the ketogenic diet shows larger numerical reductions in glycemic and weight outcomes but requires close clinical supervision due to higher adverse event rates. These findings provide updated, albeit cautious, evidence-based guidance, emphasizing that personalized dietary strategies should balance efficacy, safety, and patient preferences.

## 5 Conclusion

This meta-analysis descriptively supports the potential benefits of the Mediterranean diet, intermittent fasting, energy-restricted diet, and ketogenic diet in improving glycemic control and reducing body weight in patients with type 2 diabetes mellitus. However, due to substantial heterogeneity and prediction intervals crossing the line of no effect, the pooled estimates do not allow for definitive claims regarding relative superiority among these diets. Numerically, the ketogenic and energy-restricted diets showed larger reductions in body weight, whereas the intermittent fasting data on visceral fat reduction were inconclusive due to limited studies. The Mediterranean diet demonstrated stable improvements in glycemic control and favorable safety, while the ketogenic diet was associated with more mild adverse events and requires close clinical supervision. Because of inconsistent outcome reporting across the included trials, insulin, HOMA-IR, HDL-C, and gut microbiota data were excluded from the formal meta-analysis. Consequently, the results support tailored dietary strategies that balance efficacy, safety, and patient preferences, while emphasizing the need for cautious interpretation of indirect cross-diet comparisons.

## Data Availability

The original contributions presented in the study are included in the article/Supplementary material, further inquiries can be directed to the corresponding authors.
