# Supplementary material for: Effect of different dietary patterns on patients with type 2 diabetes: a systematic review and meta-analysis of randomized controlled trials
Source: Front Nutr. 2026 Oct 12;13:1929818. doi: 10.3389/fnut.2026.1929818 (PMC13696209; doi:10.3389/fnut.2026.1929818)
Supplement: Supplementary file 1 [file Supplementary_file_1.DOCX]

**Supplementary Table 1. Literature search strategies.**

| **Database** | **Search strategy** |
| --- | --- |
| COCHRANE | #1 MeSH descriptor: [Diabetes Mellitus, Type 2] explode all trees  #2 ("Type 2 Diabetes" or "Type 2 Diabetes Mellitus" or T2DM or "Non-Insulin-Dependent Diabetes Mellitus" or NIDDM):ti,ab,kw  #3 #1 or #2  #4 MeSH descriptor: [Diet] explode all trees  #5 ("Mediterranean Diet" or "Ketogenic Diet" or "Intermittent Fasting" or "Energy-Restricted Diet" or "Caloric Restriction" or "Dietary Patterns" or "Diet Therapy"):ti,ab,kw  #6 (Mediterranean or ketogenic or intermittent NEXT fasting or energy NEXT restrict* or calorie* NEXT restrict* or diet*):ti,ab,kw  #7 #4 or #5 or #6  #8 #3 and #7  #9 ("randomized controlled trial" or "randomised controlled trial" or RCT or placebo or "controlled clinical trial"):pt,ti,ab,kw  #10 #8 and #9 |
| PUBMED | ("Type 2 Diabetes Mellitus"[Mesh Terms] OR "Type 2 Diabetes"[Title/Abstract]) AND ("Diet"[Mesh Terms] OR "Mediterranean Diet"[Title/Abstract] OR "Ketogenic Diet"[Title/Abstract] OR "Intermittent Fasting"[Title/Abstract] OR "Energy-Restricted Diet"[Title/Abstract]) AND ("Randomized Controlled Trial"[pt]) |
| EMBASE | #1 'non insulin dependent diabetes mellitus'/exp  #2 'type 2 diabetes':ti,ab OR 'type 2 diabetes mellitus':ti,ab OR t2dm:ti,ab OR niddm:ti,ab  #3 #1 OR #2  #4 'diet'/exp  #5 'mediterranean diet':ti,ab OR 'ketogenic diet':ti,ab OR 'intermittent fasting':ti,ab OR 'energy restricted diet':ti,ab OR 'caloric restriction':ti,ab OR 'diet therapy'/exp  #6 (mediterranean OR ketogenic OR (intermittent NEXT/1 fasting) OR (energy NEXT/1 restrict*) OR (calorie* NEXT/1 restrict*) OR diet*):ti,ab  #7 #4 OR #5 OR #6  #8 #3 AND #7  #9 random*: ti,ab OR placebo*: ti,ab OR (double NEXT/1 blind*): ti,ab  #10 #8 AND #9 |

Figure 1. The risk of bias assessment results for each included study

Figure 2. Funnel plot of Waist circumference

 Figure 3. Funnel plot of TG

 Figure 4. Funnel plot of TC

Figure 5. Funnel plot of LDL-C

Figure 6. Funnel plot of HbA1C Figure 7. Funnel plot of Glycemia

Figure 8. Funnel plot of BMI Figure 9. Funnel plot of Weight

Figure 10. Sensitivity analysis of BMI

 Figure 11. Sensitivity analysis of Glycemia

Figure 12. Sensitivity analysis of HbA1C

Figure 13. Sensitivity analysis of LDL-C

Figure 14. Sensitivity analysis of TC

Figure 15. Sensitivity analysis of TG

 Figure 16. Sensitivity analysis of Waist circumference

Figure 17. Sensitivity analysis of Weight

Figure 18. Meta-regression analysis of Glycemic Indicators.

 Figure 19. Meta-regression analysis of Anthropometric Indices.

 Figure 20. Meta-regression analysis of Lipid Profile.
